# Asymmetric Dimethyarginine as Marker and Mediator in Ischemic Stroke

**DOI:** 10.3390/ijms131215983

**Published:** 2012-11-28

**Authors:** Shufen Chen, Na Li, Milani Deb-Chatterji, Qiang Dong, Jan T. Kielstein, Karin Weissenborn, Hans Worthmann

**Affiliations:** 1 Department of Neurology, Hannover Medical School, 30623 Hannover, Germany; E-Mails: sfchen_cindy@yahoo.com.cn (S.C.); Li.Na@mh-hannover.de (N.L.); Deb-Chatterji.Milani@mh-hannover.de (M.D.-C.); Weissenborn.Karin@mh-hannover.de (K.W.); 2 Department of Neurology, Huashan Hospital Fudan University, Shanghai 200040, China; E-Mail: qdong@fudan.edu.cn; 3 Center for Systems Neuroscience (ZSN), 30559 Hannover, Germany; 4 Department of Neurology, Beijing Tiantan Hospital, Capital Medical University, Beijing 10050, China; 5 Department of Nephrology and Hypertension, Hannover Medical School, 30623 Hannover, Germany; E-Mail: kielstein@yahoo.com

**Keywords:** asymmetric dimethylarginine (ADMA), symmetric dimethylarginine (SDMA), nitric oxide (NO), nitric oxide synthase (NOS), ischemic stroke

## Abstract

Asymmetric dimethylarginine (ADMA), an endogenous nitric oxide synthase (NOS) inhibitor, is known as mediator of endothelial cell dysfunction and atherosclerosis. Circulating ADMA levels are correlated with cardiovascular risk factors such as hypercholesterolemia, arterial hypertension, diabetes mellitus, hyperhomocysteinemia, age and smoking. Accordingly, clinical studies found evidence that increased ADMA levels are associated with a higher risk of cerebrovascular events. After the acute event of ischemic stroke, levels of ADMA and its analog symmetric dimethylarginine (SDMA) are elevated through augmentation of protein methylation and oxidative stress. Furthermore, cleavage of ADMA through dimethylarginine dimethylaminohydrolases (DDAHs) is reduced. This increase of dimethylarginines might be predictive for adverse clinical outcome. However, the definite role of ADMA after acute ischemic stroke still needs to be clarified. On the one hand, ADMA might contribute to brain injury by reduction of cerebral blood flow. On the other hand, ADMA might be involved in NOS-induced oxidative stress and excitotoxic neuronal death. In the present review, we highlight the current knowledge from clinical and experimental studies on ADMA and its role for stroke risk and ischemic brain injury in the hyperacute stage after stroke. Finally, further studies are warranted to unravel the relevance of the close association of dimethylarginines with stroke.

## 1. Introduction

Stroke is the primary cause of disability and the second leading cause of death worldwide [1]. Ischemic stroke represents the most frequently occurring type of stroke [2]. However, despite substantial efforts, strategies to reduce the incidence of stroke and to restrict acute brain injury after the acute event of stroke are limited. Only understanding factors involved in the pathophysiology of stroke risk and the detrimental cascade of brain injury after acute ischemic stroke, such as endothelial dysfunction and oxidative stress, can help to identify new therapeutic strategies.

In clinical and experimental studies, elevated levels of asymmetric dimethylarginine (ADMA), an endogenous inhibitor of nitric oxide synthase (NOS), are associated with endothelial dysfunction and atherosclerotic burden [3–8]. Since high ADMA levels are positively correlated with cardiovascular risk factors, the predictive value of ADMA for cardio- and recently also cerebrovascular events has been investigated [9,10]. After the acute event of stroke, ADMA levels increase depending on stroke severity [11] and might play a role in brain injury by reduction of cerebral blood flow and promotion of oxidative stress and the inflammatory reaction. This review presents the current knowledge of the role of ADMA for cerebrovascular risk and acute stroke injury.

## 2. ADMA and NOS

**Figure 1:**
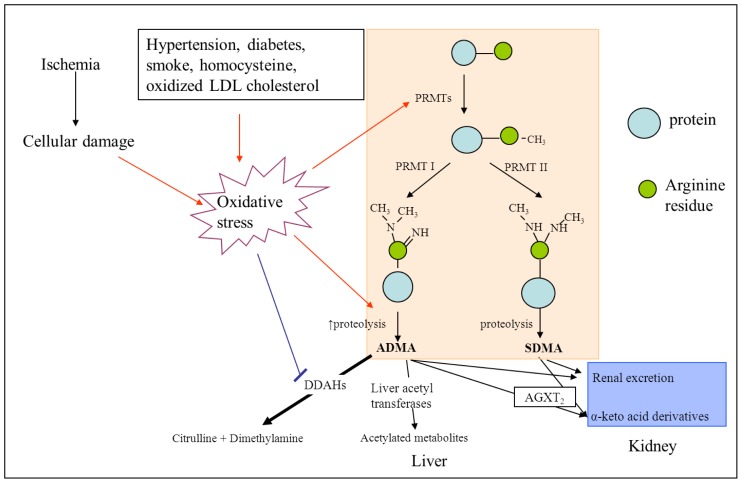
The synthesis and metabolism of ADMA and SDMA. Orange area: synthesis of ADMA and SDMA; red arrow: positive regulation; PRMTs: protein arginine methyltransferases; PRMT I: protein arginine methyltransferase type I; PRMT II: protein arginine methyltransferase type II; DDAH: dimethylarginine dimethylaminohydrolase. AGXT 2: alanine–glyoxylate aminotransferase 2. Mostly ADMA is degraded by DDAHs; to a minor degree, it is eliminated by other pathways. Acute ischemic stroke and risk factors of stroke activate oxidative stress, which could upregulate the expression of PRMTs and downregulate the activity of DDAHs, leading to increased levels of ADMA via increased production and inhibited degradation of ADMA.

ADMA is an endogenous NOS inhibitor which is produced as a by-product of protein modification processes in human cells and can be found in human plasma. It originates from the proteolysis of proteins with methylated arginine residues [12,13], which are catalyzed by protein arginine methyltransferases (PRMTs) to generate mono and dimethylarginines l-NG-monomethylarginine (l-NMMA), ADMA, and symmetric dimethylarginine (SDMA) [14] (Figure 1). According to their specific catalytic activity, PRMTs are classified into two types: type 1 catalyzes the formation of ADMA and l-NMMA, while type 2 catalyzes the formation of SDMA and l-NMMA [15]. 80% to 90% of ADMA is metabolized predominantly by dimethylarginine dimethylaminohydrolases (DDAHs), and also by alanine–glyoxylate aminotransferase 2 (AGXT2) in the kidney [16,17] or via acetylation in the liver [18]. The other smaller part is excreted renally and remains unchanged [19]. DDAH has two isoforms (DDAH-1 and DDAH-2). Their distribution has been reported in different studies. Leiper *et al.* indicate that DDAH-1 is highly expressed in epithelial cells and the only isoform found in neuronal tissues, whereas DDAH-2 is primarily present in blood vessels in diverse organs such as heart, lung, placenta and fetal tissues [20–22]. In contrast, Hu *et al.* reported that DDAH-1 is highly expressed in vascular endothelium of different organs, such as brain, kidney, lung and liver [23]. DDAH-1 degrades ADMA in the tissues [24]. DDAH activity is inhibited by oxidative stress via oxidation of a cysteine residue (Cys-249) in the catalytic site of DDAH [25,26]. In contrast, SDMA is eliminated predominantly by renal excretion but not through hydrolysis by DDAHs [12,27]. An alternative pathway for metabolization is via AGXT2 [27,28]. Accordingly, plasma SDMA has been shown to be an excellent marker of renal function [29]. NOS is an endogenous enzyme that catalyzes arginine to nitric oxide (NO) and citrulline, which is a significant process for various functions in humans, such as induction of vasodilatation, inhibition of platelet aggregation, adhesion of inflammatory cells to endothelium, and smooth muscle cell proliferation [30]. There are three isoforms of NOS in humans: nNOS, eNOS and iNOS (inducible NOS). In a stroke model of focal ischemia in rats, the expression of nNOS in neurons was shown to peak at 3 h and eNOS at 24 h after stroke onset. The peak expression of iNOS was observed at 48 h after stroke in the area of infarction [31].

l-arginine is the substrate of NOS to produce NO. As the structure of ADMA is similar to l-arginine, ADMA competes with l-arginine for NOS binding, thereby inhibiting NOS function and impairing NO formation [32]. SDMA does not inhibit NOS directly. SDMA competes with l-arginine at its transport protein human cationic amino acid transporter (hCAT)-2B. Thus, SDMA indirectly inhibits the production of NO by reduction of the availability of l-arginine to NOS [33,34]. Besides, SDMA was demonstrated to be involved in the process of inflammation in chronic kidney disease (CKD) [35], rheumatoid arthritis[36], and ischemic stroke [37]. In an *in vitro* study, SDMA enhanced the production of ROS in endothelial cells and monocytes [34,35].

## 3. ADMA and Cerebrovascular Risk

### 3.1. ADMA as a Mediator of Endothelial Dysfunction and Atherosclerosis

ADMA acts as a mediator of endothelial cell dysfunction, representing the first step in the pathophysiological process of atherosclerosis. ADMA affects the integrity and the function of the vasculature itself through diverse mechanisms [38]. In a cell culture model of human umbilical vein endothelial cells (HUVECs), it has been shown that exogenous ADMA treatment (10 μmol/L, 24 h) damaged the endothelial gap junction function, which is of importance for endothelial cell differentiation and senescence [39]. Exogenous ADMA treatment induced the adhesion of monocytoid cells to HUVECS and elevated the levels of interleukin-8 and expression of its receptor [40,41]. The process of monocyte adhesion to endothelial cells triggered by chemokines represents a decisive mechanism for the initiation of atherosclerosis [42,43]. Experimental studies suggested ADMA is involved in further steps of atherosclerosis, such as induction of smooth muscle cell (VSMC) migration, foam cell formation [44,45] and apoptosis of VMSCs and endothelial cells [41,46,47]. Endothelial progenitor cells are key players in regeneration of injured endothelium in atherosclerotic lesions. Thum and co-workers demonstrated that ADMA might suppress the differentiation and mobilization of endothelial progenitor cells in patients with coronary artery disease (CAD) [48]. Overexpression of DDAH-1 in apolipoprotein E-deficient mice reduced plaque formation in the aorta and improved endothelial function as assessed by endothelium-dependent vasodilatation [49].

### 3.2. ADMA and Its Association with Vascular Risk Factors

Elevated plasma ADMA levels have been reported in patients with vascular risk factors, such as hypercholesterolemia [9,50,51], arterial hypertension [4,9], diabetes [5], hyperhomocysteinemia [6,7,20,52], and smoking [52]. In a cross-sectional study in healthy nondiabetic subjects, plasma ADMA levels and steady-state plasma glucose representing insulin resistance were positively correlated [53]. Yoo and Lee showed a positive correlation between plasma levels of ADMA and homocysteine in 87 elderly subjects, consisting of 52 patients with history of ischemic stroke and 36 healthy controls. A homocysteine concentration above 15.0 μmol/L was independently predictive for increased ADMA levels [54]. However, in 47 patients with cerebral small vessel disease, and also in 712 participants of a health examination, circulating ADMA and homocysteine levels were not correlated [55,56].

Experimental models indicate potential mechanisms by which vascular risk factors increase ADMA. In a rat model glycated bovine serum albumin, which stands for an advanced glycated end product, inhibited DDAH activity and vascular relaxation in aortic rings [57]. However, ADMA levels had not been investigated. In human umbilical vein endothelial cells incubation with oxidized low density lipoprotein (oxLDL) (100 μmol/mL) reduced DDAH activity [58]. In human coronary artery endothelial cells, the expression of PRMTs was increased in the presence of native or oxLDL [59]. In both studies ADMA levels in the incubation media were increased.

**Figure 2:**
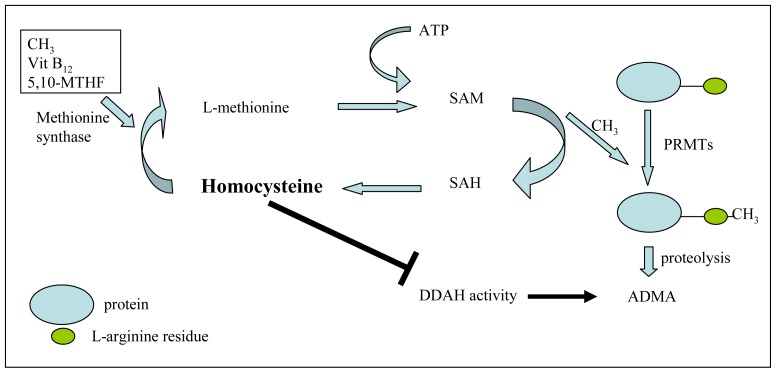
The relationship between ADMA and homocysteine. SAH: *S*-adenosyl-lhomocysteine; SAM: *S*-adenosylmethionine; CH3: methyl group; ATP: adenosine triphosphate; DDAH: dimethylarginine dimethylaminohydrolase; ADMA: asymmetric dimethylarginine; Vit B12: vitamin B12; MTHFR: methyltetrahydrofolate reductase.

It is hypothesized that hyperhomocysteinemia might increase production and decrease metabolization of ADMA (Figure 2). ADMA is methylated by PRMTs through utilization of *S*-adenosylmethionine (SAM), which serves as a universal methyl group donor for methyl transfer reactions. In the presence of vitamin B12 and 5,10-methyltetrahydrofolate, homocysteine can be remethylated to L-methionine. Therefore, methionine can be activated by adenosine triphosphate (ATP) to form SAM [59,60]. Homocysteine thus has an indirect effect upon ADMA synthesis. Furthermore, it was shown that homocysteine inhibited the activity of DDAH possibly via an oxidative reaction with its active site cysteine residue, which increases the concentration of ADMA [61]. Several studies give the evidence of the link between elevated levels of ADMA and homocysteine. Böger *et al.* showed that plasma levels of ADMA were elevated in hyperhomocysteinemia in monkeys [62]. Both a hyperhomocysteinemic diet for four weeks and an atherogenic diet that caused hyperhomocysteinemia and hypercholesterolemia for 17 months increased plasma levels of ADMA. Additionally, incubation of HUVECs with methionine or homocysteine increased the ADMA release. Selley showed that the incubation of rat neuronal granule cells with homocysteine for 24 h caused a dose-dependent decrease of DDAH activity and increase of ADMA levels in the culture medium [63].

### 3.3. ADMA and Its Association with Carotid Intima-Media Thickness

Several studies investigated the association of ADMA with the intima-media thickness (IMT) of the carotid artery. Nanayakkara and co-workers showed a significant positive correlation between plasma ADMA levels and the carotid IMT in chronic renal insufficiency patients with a creatinine clearance of 15 to 70 mL/min per 1.73 m^2^ (according to the Cockcroft-Gault equation). The correlation remained significant even after adjustment for risk factors of atherosclerosis [64]. In a large community-based sample using 2958 participants of the Framingham Heart Study (offspring cohort excluding prevalent cardiovascular disease) an independent correlation between plasma ADMA levels and the internal carotid artery IMT was detected, but not with the common carotid artery IMT. Therefore, the authors suggested that ADMA promoted atherosclerosis in a site-specific manner [65]. Plasma ADMA levels were also significantly correlated with carotid IMT in patients with subclinical carotid atherosclerosis, even those who were not taking any medication [66]. Furthermore, it was reported that plasma ADMA levels at baseline were independently predictive for carotid IMT progression over a six-year observation period in 712 subjects [67]. So far, data regarding plasma ADMA levels and restenosis after carotid endarterectomy (CEA) are lacking. In contrast to the above described data, a case series in 60 young atherosclerotic patients found an inverse correlation between plasma ADMA level and carotid IMT [68]. However, a meta-analysis in 6168 subjects from 22 studies concluded that high plasma ADMA levels were associated with carotid IMT [69].

### 3.4. ADMA and Its Association with Cardiovascular and Cerebrovascular Events

As has already been mentioned, ADMA is closely associated with vascular risk factors and promotes atherogenesis, suggesting a role of ADMA in cardiovascular events. Indeed, several studies have reported the predictive value of plasma ADMA levels for cardiovascular and cerebrovascular events [70–74]. Among healthy individuals with a low risk of cardiovascular disease according to the Systematic Coronary Risk Evaluation (SCORE) model, those with plasma ADMA levels above 0.71 μmol/L had a higher risk of cardiovascular and cerebrovascular events, as compared to those with plasma ADMA levels below [72]. Also in patients with increased vascular risk, the concentration of plasma ADMA was predictive for cardiovascular events. A prospective study in 125 patients with type 2 diabetes demonstrated a significant association of elevated plasma levels of ADMA at baseline with large vessel cardiovascular events and stroke after a 21-month follow-up. ADMA > 0.63 μmol/L had a significantly increased hazard ratio for incident cardiovascular events compared with those with ADMA ≤ 0.53 μmol/L (2.37 [95% CI 1.05–5.35], *p* = 0.038) [73]. Interestingly, in patients with end stage renal disease (ESRD), the risk of death and cardiovascular events was additionally increased when both—levels of ADMA and mediators of inflammation (C-reactive protein (CRP) or IL-6) in plasma—were elevated compared to patients with elevation of only one biomarker [71]. The authors concluded that endothelial dysfunction and inflammation may have synergic effects on cardiovascular risk and mortality in patients with ESRD. In 1011 patients who underwent elective diagnostic cardiac catheterization, elevated plasma levels of both ADMA and SDMA were independent predictors of future cardiovascular events (MACE, including myocardial infarction, stroke and death) at three years [75]. Elevated levels of ADMA in the highest quartile of 1.49–8.06 μmol/L and SDMA of 1.05–6.17 μmol/L were significant independent predictors of incident MACE (ADMA: adjusted Hazard Ratio [HR] 2.2, 95%CI 1.2 to 4.0, *p* = 0.015; SDMA: adjusted HR 2.4, 95%CI 1.2 to 4.6, *p* = 0.009). In a cohort of 3319 participants from the Framingham offspring study, plasma ADMA was independently associated with all-cause mortality but not with the rate of cardiovascular diseases, as defined by fatal or nonfatal myocardial infarction, coronary insufficiency, angina pectoris, stroke or transient ischemic attack (TIA), intermittent claudication, or heart failure after a period of 10.9 years [76]. The authors attributed the lack of association to the relatively short follow-up period in participants with only intermediate risk of cardiovascular disease. However, recently in a study in 1148 patients with stable coronary heart disease, plasma levels of SDMA, but not ADMA, were predictive for secondary cardiovascular events, while both ADMA and SDMA were predictive for all-cause mortality [77]. These results suggest SDMA to be possibly even more strongly associated with the rate of cardiovascular events than ADMA. Finally, factors beyond direct NOS inhibition should be considered to explain the association between dimethylarginines and cardiovascular events. The Framingham Heart Study showed that women with early natural menopause (<42 years) had an increased risk of ischemic stroke [78]. Of note, a study particularly targeting the female gender, which is a 24-year follow-up cohort from the Population Study of Women in Gothenburg (*n* = 880), showed that plasma ADMA levels served as an independent predictive marker for myocardial infarction and stroke in women. An increase of 0.15 μmol/L (1SD) in plasma ADMA levels was associated with an approximate 30% increase in incidence of myocardial infarction and stroke for woman [72]. Therefore, individuals who are at higher than the expected risk based on the SCORE and Framingham systems could be identified. These studies provided evidence for gender differences in ADMA levels.

An increasing number of clinical studies investigated the association of high plasma ADMA levels with cerebrovascular disease [10,54–56]. Among 2013 subjects of the Framingham offspring cohort who had no prevalent stroke, dementia, or other neurologic illness which could affect magnetic resonance imaging (MRI) findings, it was observed that silent brain infarcts in MRI occur more frequently in subjects in the upper three age-specific quartiles of plasma ADMA concentrations compared to the lowest quartile. This association remained significant even after adjusting for age, sex, cardiovascular risk factors, statin and antithrombotic therapies, and concentrations of creatinine and homocysteine [10]. Also Notsu *et al.* demonstrated in a sample of 712 subjects experiencing a health examination that a low l-arginine/ADMA ratio in plasma was independently associated with microangiopathy-related cerebral damage, which had been defined as a composite of lacunar infarction and white matter hyperintensity [55]. In 16 patients with subcortical infarcts and leukencephalopathy (CADASIL) an inverse correlation was found between the L-arginine/ADMA ratio in plasma and T2 weighted lesion volumes in MRI, indicating endothelial dysfunction in these patients [79]. In a Korean study, elder patients with a history of stroke showed significantly higher plasma ADMA levels than their age-matched healthy controls. This study demonstrated that in the subgroup of patients with recurrent stroke plasma ADMA levels were even higher than in the subgroup of patients with first ever stroke (2.28 ± 1.63 μmol/L for recurrent stroke, and 1.46 ± 0.77 μmol/L for first ever stroke) [54]. Of note, Ding *et al.* identified a novel four-nucleotide (−396 4N) deletion-insertion (del-ins) polymorphism in the DDAH-1 promoter region by resequencing the DDAH-1 gene in 49 individuals from the Han Chinese population [80]. *In vitro*, the DDAH-1 transcriptional activity was significantly reduced for the −396 4N ins allele. Subjects with the −396 4N ins variant had lower DDAH-1 mRNA expression in the lymphocytes and higher plasma ADMA levels than those with the −396 4N del allele. Moreover, the association of the −396 4N del-ins polymorphism with stroke was investigated in two cohorts of stroke patients of Han Chinese (discovery sample: 1388 cases and 1027 controls; replication sample: 961 cases and 822 controls). The −396 4N ins allele was associated with increased risk for atherothrombotic stroke in both samples. However, in the discovery sample, but not in the replication sample, an association with increased risk for lacunar stroke, hemorrhagic stroke and overall stroke was identified. The study of Lu *et al.* that investigated six single nucleotide polymorphisms (SNPs) in DDAH-1 (rs233112, rs1498373, rs1498374, rs1403956, rs1241321 and rs 587843) in a cohort of 309 type 2 diabetic patients and 505 non-diabetic controls showed controversial results [81]. Although the homozygous AA genotype in SNP rs1241321 was associated with a reduced risk for all cause mortality and the combined endpoint of cardiovascular death, nonfatal myocardial infarction and stroke, the other four of SNPs but not SNP rs1241321 were associated with ADMA plasma levels. These findings imply that the association between DDAH polymorphism circulating ADMA levels and stroke incidence remains far from clear. The intracellular ADMA concentrations might be the key for interpretation.

### 3.5. ADMA as Pharmacological Target for Treatment of Cardiovascular Risk

Vascular risk factors, which are associated with elevated circulating levels of ADMA, are modifiable by lifestyle or pharmacological treatment. Although antihypertensive drugs via renin-angiotensin system (RAS) blockade have been consistently demonstrated to reduce ADMA levels, the impact of other drug classes on ADMA levels remains unclear (Maas 2005 [82], Caplin *et al* 2012 [28]). In particular, in studies of statins (HMG-CoA reductase inhibitors), the treatment effects on ADMA levels were less clear. After a six-week treatment with rosuvastatin (10 mg/day) in hypercholesterolemic patients, plasma ADMA levels were reduced [83]. Also in 56 ischemic stroke patients with LDL-cholesterol levels higher than 140 mg/dL, who were included in the study at least three months after the event of stroke and had no history of statin use, ADMA levels were significantly reduced after treatment with pravastatin, fluvastatin, pitavastatin or atorvastatin for more than three months [84]. In contrast to this, in several other studies, statin treatment had no impact on ADMA levels [85–87].

Data from the Framingham Heart Study showed that women with early natural menopause (<42 years) had an increased risk of ischemic stroke [78], suggesting a crucial role of estrogen in vascular protection. Clinical and experimental data indicate that estrogen influences the DDAH/ADMA/NO pathway. In a multicenter, placebo-controlled, double-blind study, in 152 postmenopausal women treatment with estradiol lowered plasma ADMA levels compared with the placebo group [88]. Given that plasma arginine levels declined consistently with plasma ADMA levels in the study, it is unknown whether the reduction of ADMA by estrogen would bring an increased NO production. Of note, in HUVECs exposed to either oxidized LDL cholesterol or native LDL, an effect of estradiol on NO restoration via the DDAH/ADMA/NO pathway was found [89].

Also, the regulation of DDAH has been investigated as a therapeutic target in cardiovascular disease [90]. However, further studies are needed to elucidate whether the occurrence of cardiovascular events might be reducible by targeting ADMA.

## 4. The Role of ADMA in Acute Ischemic Stroke

### 4.1. Increase of ADMA after Acute Ischemic Stroke and Its Association with Outcome

While the role of ADMA for stroke risk becomes more and more evident, the role of ADMA in acute stroke injury remains unclear. After the event of stroke, cellular damage and proteolysis induce oxidative stress in the region of the lesion (for a review see Chen *et al.* 2011 [91]). Different studies showed that oxidative stress causes an increase in the expression of PRMTs and a decrease of DDAH activity [25,92]. Both mechanisms—one by increased production, the other by decreased metabolization—result in elevated intracellular levels of ADMA (Figure 1). Thus, also circulating ADMA levels might increase after acute stroke. Several studies investigated ADMA levels after acute stroke and their association with clinical outcome. Brouns *et al.* investigated ADMA levels in the cerebrospinal fluid (CSF) of 88 patients with hyperacute stroke or TIA [93], inasmuch as CSF was drawn within 24 h after stroke onset. ADMA levels in CSF significantly differed depending on stroke severity at admission and stroke outcome at three months, but not on stroke subtypes according to the Trial of Org 10172 in Acute Stroke Treatment (TOAST) criteria [94]. Patients with higher National Institutes of Health Stroke Scale (NIHSS) Score at admission and poor outcome at 90 days as measured by modified Rankin Scale (mRS) had significantly higher CSF ADMA levels. The authors suggested the elevated CSF ADMA concentration in severe strokes and the association of CSF ADMA with outcome to result from increased cellular damage and proteolysis after stroke onset [93]. However, increase of ADMA may also be involved in secondary stroke injury due to inhibition of NOS and subsequent low NO concentrations (see section 3.2). In contrast, some studies showed that ADMA levels differed on the stroke subtypes. In a German study, Scherbakov *et al.* found plasma concentrations of ADMA increased in 60 acute stroke patients, predominantly in patients with cardioembolic or large-artery atherosclerotic infarct etiologies (0.591 ± 0.79μmol/L and 0.599 ± 0.91 μmol/L, respectively) [95]. In a Swedish study, in 363 stroke patients, the serum concentrations of ADMA were increased in the subgroups with TIA or cardioembolic stroke (0.54 ± 0.05 μmol/L and 0.55 ± 0.08 μmol/L, respectively), but not in non-cardioembolic stroke and hemorrhagic stroke (0.51 ± 0.07 μmol/L and 0.51 ± 0.11 μmol/L, respectively) [96]. So far, these differences could not be explained pathophysiologically. However, Scherbakov and colleagues also indicated that their subgroup analysis should be observed with caution due to low case numbers. A contradictory result was found in a study with 238 Hispanic patients. Here, plasma ADMA concentrations after acute stroke did not differ from those in healthy controls [97]. The authors indicated that differences between the studies might be due to different genetic, socioeconomic and nutritional factors. As a limitation, stroke subtypes for patients were not indicated in the study. Importantly, the different findings about plasma ADMA levels in acute stroke could also depend on the time point of blood withdrawal after stroke onset. Therefore, our group recently investigated the time course of plasma ADMA levels in 67 acute ischemic stroke patients. We showed that plasma ADMA concentrations increased in a temporal pattern in dependency on stroke severity after acute stroke. In patients with a favorable outcome, plasma ADMA levels increased during the first day after stroke and decreased again after day 3, while in patients with unfavorable outcomes, plasma ADMA increased until day 3 and remained stable until day 7 before it decreased again [11].

Plasma ADMA levels in the acute stage of stroke independently predicted adverse outcome at 90 days. ADMA levels ≥0.566 μmol/L at day 3 and ≥0.530 μmol/L at day 7 after symptom onset were independent predictors of an unfavorable outcome [11]. These data together with the findings from various studies reporting an independent association between ADMA and the incidence of stroke indicate that ADMA might be a marker specific for ischemic stroke.

Interestingly, plasma SDMA has been also shown to be increased after ischemic stroke and might be associated with outcome. In our study, in patients with unfavorable outcomes, increased plasma SDMA levels were observed from 24 h until three days after stroke. Of note, plasma SDMA levels at 24 h could independently predict adverse 90-day outcome, as well [11]. Schulze *et al.* showed in a 7.4-year follow-up study among 394 stroke patients that plasma ADMA and SDMA concentrations were associated with all-cause mortality during the follow-up. After adjusting for age, stroke subtype, previous stroke or TIA, atrial fibrillation and estimated glomerular filtration rate (eGFR), only plasma SDMA levels remained an independent predictor of all-cause mortality [37]. Recently, Lüneburg *et al.* showed that plasma SDMA predicted detrimental outcome (death, recurrent stroke, myocardial infarction, re-hospitalization) in the following 30 days after ischemic stroke onset, in patients who were recruited immediately after admission to the emergency unit. However, when adjusted for age, eGFR, NIHSS at admission, CRP and Hct (haematocrit), the impact of SDMA on detrimental outcome was lost [98]. Given that the longitudinal cohort study could give more convincing evidence than the short-term study, and different eGFR equations to evaluate kidney function were used, these results are not directly comparable.

### 4.2. Involvement of ADMA in Brain Injury after Stroke

**Figure 3:**
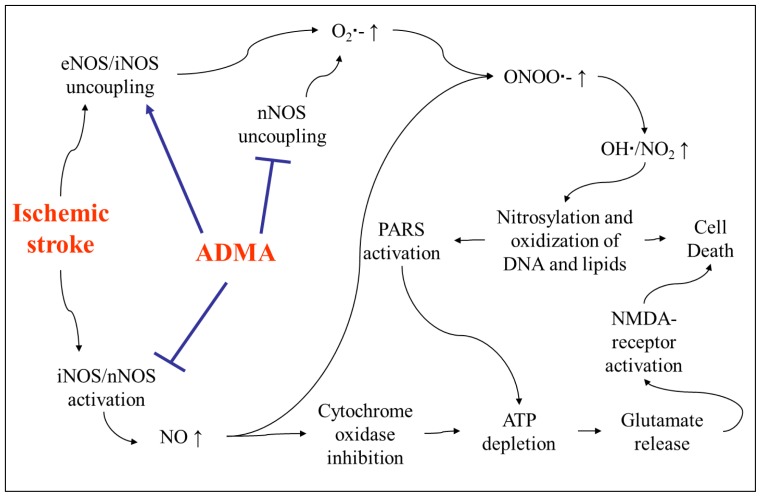
ADMA-NOS-pathway in ischemic stroke. ONOO· ^−^ : peroxynitrite; OH·: hydroxy radical; NO_2_: nitrogen dioxide; O_2_· ^−^ : oxygen radicals; NMDA: *N*-methyl-d-aspartate; PARS: poly ADP-ribose synthetase.

ADMA increase and its association with stroke outcome might represent not only a marker but also a mediator for brain injury after acute stroke. One effect of ADMA might be the restriction of cerebral blood flow (CBF). ADMA could play an important role on cerebrovascular compliance and CBF in resting conditions and after the acute stroke by inhibition of NOS, since NO is the most important endogenous vasodilator for regulation of CBF [99]. These thoughts are corroborated by data from animal models. Treatment with ADMA significantly constricted the diameter of the basilar artery in rats and the diameter of cerebral arterioles in rabbits. These effects could be prevented by l-arginine administration [100]. Dayoub and his group reported that overexpression of DDAH-1 in transgenic (TG) mice increased the relaxation responses of carotid arteries to acetylcholine (ACh), and these responses could be partially affected by ADMA administration. In this TG mouse model, an enhanced relaxation response was also observed in cerebral arterioles. However, additional ADMA could not abolish the enhancement. In contrast, additional ADMA treatment (10 μmol/L, 20min) could reduce responses of cerebral arterioles to ACh by about 70% in wild type (WT) mice [101]. Hence, the authors concluded that overexpression of DDAH represents a potential regulator of cerebral perfusion. The importance of ADMA for cerebral perfusion was also observed in humans. In rings of human middle cerebral artery from 26 autopsies, exogenous ADMA caused concentration- and endothelium-dependent contractions, which could be prevented by l-arginine [102]. Kielstein *et al.* showed a decrease of the total cerebral perfusion and an increase of the augmentation index—a measure for the arterial stiffness—in 20 healthy subjects after infusion of ADMA (0.10mg/kg body weight per minute over a period of 40 min). Of note, the systemic blood pressure was not affected [103]. Thus, it can be expected that increased plasma ADMA affects the blood supply of the brain after stroke onset. Accordingly, ADMA may impair perfusion in the penumbra and aggravate the brain tissue injury after acute ischemic stroke. Of note, Leypoldt *et al.* reported that in a mouse model of temporal middle cerebral artery occlusion (tMCAO) infarct size between DDAH-1 TG mice and WT littermates did not differ [104]. However, DDAH activity in the brain of DDAH-1 TG mice was not different from WT animals and also ADMA brain tissue levels were stable. The authors suggested that these findings might be explained by high basal cerebral DDAH activity, which is not further increasable by TG overexpression of DDAH in the used model. In contrast to the results in patients, in a rat model of tMCAO, ADMA levels in CSF were lowered, while they were unchanged in serum and brain [105].The other effect of ADMA on acute ischemic stroke might be its interaction with the CNS. A cell culture study showed that ADMA inhibited NO production from nNOS and protected neurons from overexpressed NO mediated injury [106]. Therefore, ADMA might have a protective effect on the central nervous system (CNS). However, there is accumulated evidence suggesting a link between ADMA, oxidative stress and inflammation after acute stroke, which is a vicious circle to the brain. On the one hand, oxidative stress elevates ADMA levels through activation of PRMTs and inhibition of DDAH activity [14,92]. On the other hand, ADMA acts as a mediator of oxidative stress via uncoupling of eNOS and iNOS leading to massive superoxide (O_2_· ^−^ ) production [92,107]. However, direct evidence that ADMA uncouples the different NOS in the cerebral circulation is still missing. Additionally, massively increased NO produced by iNOS and nNOS after ischemic stroke acts as a neurotoxic molecule [108–110]. NO largely reacts with O_2_· ^−^ . Thereby the most toxic compound peroxynitrite (ONOO· ^−^) is formed [31], leading to neuronal cell death. In the condition of cofactor tetrahydrobiopterin (BH_4_) or arginine depletion which has been observed after acute stroke, O_2_· ^−^ becomes the main product of nNOS instead of NO. Of note, ADMA can attenuate this process in a dose dependent way. In the absence of l-arginine ADMA levels in a concentration of 1 μM or higher markedly inhibited nNOS-derived O_2_· ^−^ generation [111]. However, further studies are needed to clarify the role for ADMA in inhibition of NOS isoforms after stroke. Expression of NOS isoforms in cerebral ischemia differs temporally and spatially. The effects of NOS are dependent on the amount produced [112]. In addition, the inhibition of iNOS and nNOS is suggested to be neuroprotective and eNOS inhibition might reduce CBF after brain injury. Therefore the effects of ADMA—which acts as a nonselective NOS inhibitor and a mediator of oxidative stress via uncoupling of iNOS and eNOS—may be multiplex, either detrimental or beneficial (Figure 3). Moreover, although ADMA synthesized by endothelial cells has been shown to regulate NO production in macrophages in a cell culture model [113], so far, direct evidence is lacking if endothelial ADMA might also influence NO production in neurons. All these aspects may complicate the therapeutic strategy targeting the NO-DDAH-ADMA pathway in ischemic stroke. Isoform-specific inhibition of DDAHs has shown to be a promising experimental strategy in models of vasodilatory septic shock [114]. Further experimental studies using this strategy might be helpful to elucidate the current unclear situation.

Moreover, in cell culture models, ADMA promotes inflammation via increased production of pro-inflammatory cytokines [115,116]. In stroke, this would be of importance, since the acute inflammatory reaction after the acute event triggers secondary brain injury and thereby is linked with clinical outcome [117]. It was reported that ADMA induced tumor necrosis factor-alpha (TNF-alpha) production via ROS/NF-kappaB dependent pathway in monocytes [115]. In endothelial cells, ADMA enhanced the concentrations of TNF-alpha and soluble intercellular adhesion molecule-1, the activity of NF-kappaB, and the phosphorylation of mitogen-activated protein kinases [116]. In another study, exposure of polymorphonuclear neutrophils (PMN) to ADMA enhanced PMN adhesion to endothelial cells and degranulation [118]. Several studies using human DDAH-1 TG mice demonstrated that the lowering of ADMA through DDAH modulation in conditions of acute or chronic inflammation might be beneficial [118–121]. In human DDAH-1 TG mice leukocyte activation and concomitant release of myeloperoxidase (MPO) induced by long-term ADMA infusion were attenuated by overexpression of DDAH-1 [118]. DDAH-1 overexpression accelerated endothelial regeneration and lowered the vascular inflammatory infiltration in a lesion of the left femoral artery [120]. In human DDAH-1-TG mice after heterotopic heart transplantation, myocardial generation of superoxide anion and TNF-alpha, as well as luminal narrowing and intima–media ratio of affected vessels were reduced [119]. Myocardial reperfusion injury in a model of occlusion and reopening of the left coronary artery was reduced by 40–50% in human DDAH-1 TG mice, via decreased production of ADMA, which mediated eNOS activity and phosphorylation, expression of adhesion molecules, and leukocyte activity [121]. However, thus far, studies which investigate the possible link between ADMA and inflammation in the acute pathology after ischemic stroke are missing.

In comparison to ADMA, studies investigating the pathophysiological role of SDMA are rare. Experimental data showed that SDMA in concentrations of 1–10 mmol/L indirectly interferes with NO synthesis through impairment of intracellular l-arginine uptake and decrease of renal tubular arginine absorption [33,122]. However, these results might not reflect the physiological effects of SDMA *in vivo* due to the concentrations used. There are very few *in vitro* studies using concentrations of SDMA more similar to those *in vivo*. It was reported that SDMA (2–100 μmol/L) inhibited NO synthesis dose dependently and induced ROS production in endothelial cells [34]. In monocytes SDMA (6.1 μmol/L) stimulated ROS production via modulation of store-operated calcium channels [123]. These studies imply that SDMA might contribute to the brain injury after ischemic stroke via pro-inflammatory effects and inhibition of NO synthesis in endothelial cells. It was reported that SDMA enhanced activation of NF-kappaB and expression of TNF-alpha and IL-6 in monocytes [35]. Elevated plasma SDMA levels were found to correlate with inflammatory markers, such as TNF-alpha, high sensitivity CRP and Interleukin-6 (IL-6) in chronic kidney disease (CKD) patients [35,124,125]. In patients following ischemic stroke plasma SDMA levels were shown to correlate with plasma CRP levels [37]. Therefore, SDMA might contribute to the brain injury after ischemic stroke via pro-inflammatory effects and inhibition of NO synthesis in endothelial cells.

## 5. Conclusions

ADMA, an endogenous nonselective NOS inhibitor, is closely related to the development of endothelial dysfunction and atherosclerosis, and is suggested to be involved in an increased risk of cardiovascular diseases and stroke. By its pathophysiological role, ADMA might have additional predictive value for stroke beyond traditional risk factors, which could guide new treatment approaches for stroke prevention. Additionally, accumulated clinical studies indicated that high ADMA concentrations early after stroke might predict stroke outcome. However, the role of elevated ADMA levels for brain injury after acute ischemic stroke remains unclear. Further studies are warranted to elucidate the involvement of ADMA but also of its analog SDMA in the pathological process after ischemic stroke.
